# Editorial: Neoadjuvant Therapy in Rectal Cancer: Response Prediction and Organ Preservation Strategies

**DOI:** 10.3389/fonc.2022.944741

**Published:** 2022-06-09

**Authors:** Samuel Aguiar, Silvia Regina Rogatto

**Affiliations:** ^1^Colorectal Cancer Reference Center - AC Camargo Cancer Center, Sao Paulo, Brazil; ^2^Department of Clinical Genetics, University Hospital of Southern Denmark, Vejle, Denmark; ^3^Institute of Regional Health Research, University of Southern Denmark, Odende, Denmark; ^4^Danish Colorectal Cancer Center South, Vejle, Denmark

**Keywords:** rectal cancer, neoadjuvant (chemo)radiotherapy, biomarkers, prediction of response, prognostic factor, predictive factor, organ preservation

Colorectal cancer is one of the most incident and lethal cancers worldwide, and rectal location represents about one third of all cases. The treatment of rectal cancer remains challenging and changing over the years and is a model of multidisciplinary approach. Currently, the treatment planning of rectal cancer is not acceptable without a perfect harmony among imaging, endoscopy, pathology, surgery, radiation, and clinical oncology. Since the consolidation of the neoadjuvant chemoradiation and the techniques of total mesorectal excision almost 20 years ago (1), relevant changes have emerged in the last years. The watch and wait (WW) strategy, proposed by Habr-Gama and her group since the end of the 20^th^ century, has been validated by several independent cohorts, from different countries, and now is part of any clinical practice guideline (2). Two different modalities of preoperative radiation, short-course and long-course, as well as the total neoadjuvant chemotherapy, also plays important roles in the treatment decision-making (3, 4). Nowadays, we need to decide which patient needs to be operated upfront or should not be ever operated (WW); which patient really has benefit with neoadjuvant chemotherapy or can be spared of oxaliplatin-related adverse effects; or which patient can even receive chemotherapy alone, without radiation, as neoadjuvant treatment; or which patient can even be treated only with immunotherapy (5). Pathological complete response (cPR) after neoadjuvant treatment is one of the most important outcomes in rectal cancer, but can only be identified after radical surgery. Complete clinical response (cCR) is the endpoint to be achieved, as it is the key for non-operative strategies. Unfortunately, current clinical tools still failure to classify a cCR as a real cPR. Prediction of therapy response, for better and safer selection of patients to different arms of treatment, has been a rich field for clinical, translational and basic research, and stimulated us to propose this Research Topic.

In this Research Topic, interesting findings were reported aiming to improve the prediction of response to neoadjuvant therapies. By using only clinical and imaging data, Liu et al. pointed out for low performance of both multi-slice computed tomography (MSCT) or magnetic resonance imaging (MRI) in predicting ypT0-T1. On the other hand, Pyo et al. show an accuracy of 84.8% in predicting cPR with a model that combines CEA level, MRI and PET/CT for response assessment after neoadjuvant chemoradiation. Based on these three methods, the authors propose a nomogram for selecting patients for organ preservation.

Immunohistochemical analyses from pre-treatment tumor samples also appear as prognostic and predictive factors. Focusing on tumor immune microenvironment, Yang et al. found that high levels of cytotoxic T lymphocytes were significantly associated with pCR, whereas tumor-associated macrophages in tumor tissue were associated with poor response. Wang et al. analyzed immunohistochemical expression of RUNX3 and EZH2 proteins in pre-treatment samples from 80 patients. High expression of RUNX3 and low expression of EZH2 were significantly associated with good tumor regression (TRG grade 0/1). Moreover, high expression of RUNX3 in pre-treatment tumor samples was strongly associated with very high rates of disease-free and overall survival. Immunohistochemical markers have special advantages of the easy validation and relatively low costs.

Biomarkers associated with irradiation response were also explored. Wu et al. used miRNA expression analysis in patient-derived xenografts (PDX) paired with pre-storage specimens from the same patient. They found four miRNAs (miRNA-552-3p, miRNA-96-5p, miRNA-182-5p, and miRNA-183-5p) significantly up-regulated in irradiation-resistant tissues, but only the miRNA-96-5p was positively correlated with the resistance to radiation. Functional assays supported that the *GPC3* gene is directly regulated by miRNA-96-5p and is a plausible mechanism associated with irradiation resistance in rectal cancer cells and related to the alterations of the Wnt/b-catenin signal transduction pathway. Wei et al. explored immune-related genes differentially expressed using gene expression datasets (GEO and TCGA) of locally advanced rectal cancer (LARC). Based on this gene list, they constructed a response-related prediction model and a competitive endogenous RNA network. Among the results, they found that hsamir-107 and the lncRNA WDFY3-AS2 were associated with survival and are potential prognostic markers or therapeutic targets for LARC patients. The authors built a prognostic risk score model with a good predictive value for the response to chemotherapy. Iseas et al. integrated several factors (mismatch-repair deficiency markers, HER2, CDX2, PD-L1 expression, and CD3−CD8+ tumor-infiltrating lymphocyte) with clinical data and targeted DNA sequencing of non-metastatic rectal cancer patients. Interestingly, the authors found two distinct groups of patients showing a synergic role of *KRAS* and *TP53* mutational status and tumor immune infiltrate. High neutrophil-platelet scores and *KRAS* mutated cases were found as independent predictive factors and associated with a worse response to treatment. The role of local microbiota in modifying the response to neoadjuvant therapy was investigated by Takenaka et al. in 44 patients prospectively recruited. In this South American prospective cohort, from Brazil and Argentina, the authors identified a group of bacteria, *Enhydrobacter*, *Paraprevotella*, and especially *Finegoldia*, that were significantly associated with poor response to therapy. These findings can open a window for increasing response rates by modulating intestinal microbiota prior to neoadjuvant treatment, a strategy that could be tested in future clinical trials.

This Research Topic explored a plethora of strategies focusing on rectal cancer and response to therapy that highlighted new markers or targets for therapy, that improve our understanding in this exciting area. Unfortunately, we still could not identify one or a group of predictive biomarkers that can be finally incorporated in clinical practice. The findings described here have potential applications and are strong candidates to be validated in larger cohorts of patients or in future clinical trials. The prediction of response to therapy in rectal cancer remains challenging.

## Author Contributions

SA: editor of the Research Topic; writer of the editorial SR: editor of the Research Topic; writer of the editorial. All authors contributed to the article and approved the submitted version.

## Funding

The Danish Colorectal Cancer Center South and Research Council Lillebaelt Hospital, Denmark.

## Conflict of Interest

The authors declare that the research was conducted in the absence of any commercial or financial relationships that could be construed as a potential conflict of interest.

## Publisher’s Note

All claims expressed in this article are solely those of the authors and do not necessarily represent those of their affiliated organizations, or those of the publisher, the editors and the reviewers. Any product that may be evaluated in this article, or claim that may be made by its manufacturer, is not guaranteed or endorsed by the publisher.
